# *Alu* retrotransposons and COVID-19 susceptibility and morbidity

**DOI:** 10.1186/s40246-020-00299-9

**Published:** 2021-01-04

**Authors:** Manci Li, Luca Schifanella, Peter A. Larsen

**Affiliations:** 1 Department of Veterinary and Biomedical Sciences, University of Minnesota, St. Paul, MN 55108 USA; 2 Department of Surgery, Division of Surgical Outcomes and Precision Medicine Research, University of Minnesota Medical School, Minneapolis, MN 55455 USA

**Keywords:** *ACE*, *PGR*, *PLAT*, *F13B*, Human evolution, SARS-CoV-2, COVID-19

## Abstract

SARS-CoV-2 has spread rapidly across the world and is negatively impacting the global human population. COVID-19 patients display a wide variety of symptoms and clinical outcomes, including those attributed to genetic ancestry. *Alu* retrotransposons have played an important role in human evolution, and their variants influence host response to viral infection. Intronic *Alu*s regulate gene expression through several mechanisms, including both genetic and epigenetic pathways. With respect to SARS-CoV-2, an intronic *Alu* within the *ACE* gene is hypothesized to be associated with COVID-19 susceptibility and morbidity. Here, we review specific *Alu* polymorphisms that are of particular interest when considering host response to SARS-CoV-2 infection, especially polymorphic *Alu* insertions in genes associated with immune response and coagulation/fibrinolysis cascade. We posit that additional research focused on *Alu*-related pathways could yield novel biomarkers capable of predicting clinical outcomes as well as patient-specific treatment strategies for COVID-19 and related infectious diseases.

**Supplementary Information:**

The online version contains supplementary material available at 10.1186/s40246-020-00299-9.

## Introduction

COVID-19 (coronavirus disease 2019) is a zoonotic infectious disease that primarily affects the respiratory system, caused by SARS-CoV-2 (severe acute respiratory syndrome coronavirus 2). As of 28 December, 2020, SARS-CoV-2 has infected 79,673,754 people and is responsible for 1,761,381 deaths globally (World Health Organization; www.who.int). Pneumonia and acute respiratory distress syndrome (ARDS) are considered as the severe pulmonary manifestation of COVID-19 [1]. As SARS-CoV-2 spreads rapidly across the world, clinicians have observed a wide variety of extrapulmonary manifestations involving, at variable frequencies, multiple organs and biological systems in the human body, such as the cardiovascular, gastrointestinal, and nervous systems [1]. SARS-CoV-2 utilizes the ACE2 (angiotensin-converting enzyme 2) as a receptor for viral entry into host cells through engagement with its spike protein. The affinity of the spike protein to ACE2 is associated with the transmissibility of the virus [2] and expression levels of ACE2 are associated with susceptibility to SARS-CoV-2 [3]. The entry process requires the priming of the viral spike protein in part by TMPRSS2, a cellular surface serine protease upregulated by androgen [4]. Like SARS-CoV, SARS-CoV-2 likely downregulates ACE2 as it gains cell entry, this may directly disrupt the local equilibrium between the ACE-centered proinflammatory arms and ACE2-mediated anti-inflammatory pathways [5, 6].

General mechanisms of multi-organ damage and multi-system dysfunction observed in COVID-19 are potentially due to a combination of ubiquitous expression of ACE2 throughout the body, and the critical role ACE2 plays in maintaining the homeostasis of the renin-angiotensin-aldosterone system (RAAS) [1]. The relatively high expression of ACE2 in the cardiovascular system, especially endothelial cells of blood vessels, contributes to the thromboinflammation and hypercoagulative state prevalent in COVID-19 [7, 8]. As with many other infectious diseases, there are differential clinical manifestations and host responses among COVID-19 patients, including those having links to both biological sex and race. Preliminary analysis demonstrated that the case fatality rate is higher in males when compared to females in most countries by 1.7 times on average [9]; however, more studies are needed to confirm this observation. In the USA, while non-Hispanic black Americans comprise approximately 13.4% of the population (https://www.census.gov/quickfacts/fact/table/US/PST045219, accessed on November 30, 2020), they account for approximately 19% of deaths (https://covid.cdc.gov/covid-data-tracker/#demographics, accessed on November 18, 2020). Cultural and social determinants of health and disease are largely at play in this pandemic. Exploring the potential biological factors in addition to socioeconomic factors, such as genetic polymorphisms that underlie COVID-19 susceptibility and morbidity, is crucial in the context of disease risk, and the identification of novel personalized therapeutics.

Currently, a number of single-nucleotide polymorphisms (SNPs) in several genes and chromosome regions, such as *ACE2*, *TMPRSS2*, *HLA* alleles, and locus 3p21.31, have been identified to be associated with the susceptibility and outcome of COVID-19 by GWAS and exome sequencing as recently reviewed by Anastassopoulou et al. and Brest et al. [10, 11]. The initial whole-genome sequencing was completed in China and confirmed the association of *HLA* alleles and *TMEM189-UBE2V1* region with the development of severe outcomes of COVID-19 in the Chinese population [12]. While GWAS has proven significant in identifying high-risk alleles, the design of many GWAS studies (i.e., using population-based controls instead of individuals with confirmed mild or asymptomatic COVID-19) and the intrinsic limitations of GWAS require additional efforts focused on the exploration of the biological basis for differential host response to SARS-CoV-2 [13].

*Alu* retrotransposons are primate-specific transposable elements that amplify in the genome through an RNA intermediate [14]. These ~300 bp long mobile genetic elements compose over 10% of the human genome [15]. *Alu* elements have been extremely helpful in elucidating primate evolution and demography, especially with respect to human genetic ancestry and migration patterns [16, 17]. Along the same line, numerous *Alu* elements are associated with SNPs that are linked to human diseases and physiological traits, including COVID-19 [18–20]. Indeed, an intronic *Alu* polymorphism in the *ACE* gene has already been associated with both susceptibility and morbidity of SARS-CoV-2 infection [19, 20]. A growing body of literature suggests that *Alu*s play key regulatory roles in modulating gene expression involved in a wide range of physiological processes [21–23]. Knowing this, and given close ties with human demographics, it is worth considering the role *Alu* polymorphisms might have in the host response to SARS-CoV-2 infection, especially polymorphic *Alu* insertions in genes that are central to immune response and the coagulation/fibrinolysis cascade, such as *ACE*, *PLAT*, and *F13B*, as well as *PGR* which encodes for the progesterone receptor.

Here, we briefly summarize and examine known *Alu* polymorphisms in *ACE*, *PGR*, *PLAT*, *F13B* regarding their biological functions, roles in disease, and implications for COVID-19. The genomic information of these polymorphic *Alu*s is provided in Table 1. Our intent is to increase awareness and spur downstream research focused on the genetic and epigenetic factors that might help explain COVID-19 susceptibility and morbidity. Such information may be critical for the development of novel biomarkers or personalized therapeutics for COVID-19 and related infectious diseases.

**Table 1:** Genomic information of polymorphic *Alu*s in *ACE*, *PGR*, *PLAT*, and *F13B*

Gene with polymorphic *Alu*	Reference SNP cluster ID	Genomic location	Average frequency of insertion^b^			
			African	European	His/Latino	Asian
*ACE*	rs4343^a^	Chr17:63488670	0.35	0.42	0.47	0.60
*PGR*	rs1042838^a^	Chr11:101062681	0.018	0.17	0.12	0.034
*PLAT*	rs4646972	Chr8: 42039905	0.31	0.52	0.47	0.50
*F13B*	rs70942849	Chr1:195278188	0.14	0.40	0.47	0.73

^a^The *Alu* insertion itself does not have a rs number but it is in a complete LD with the presented rs number

^b^Calculated from ALFRED (https://alfred.med.yale.edu/alfred/)

## Methods

### Selection of polymorphic *Alu* elements

Cardiovascular complications are common in severe cases of COVID-19 and have been ascribed to disrupted homeostasis in the renin-angiotensin-aldosterone system, the coagulation cascade, and the fibrinolysis system [1, 24]. With previous knowledge and clinical experience, we recognized that there are polymorphic *Alu*s in the *ACE*, *PLAT*, and *F13B* genes that have either been confirmed or are hypothesized to influence the regulation of their corresponding products, especially in the context of cardiovascular disease and viral infection. Additionally, we include the polymorphic *Alu* in the *PGR* gene due to the known clinical effects of progesterone in regulating inflammation and cardiovascular health as well as the administration of progesterone in an ongoing COVID-19 clinical trial (ClinicalTrials.gov). We searched PubMed for all English-language original articles, reviews, and meta-analyses reporting on associations between the polymorphic *Alu*s in the four genes and cardiovascular diseases as well as susceptibility and potential effects on outcomes of viral infection. We also searched for ongoing or completed COVID-19 clinical trials associated with ACE inhibitor, ACE receptor blocker, progesterone, and tPA at ClinicalTrials.gov.

### Calculating the rate of polymorphic *Alu* insertions

All data of polymorphic *Alu* insertions were downloaded from the ALFRED database, except for Hispanic/Latino data of *PGR*, which was compiled from various sources including ALFRED [25–27] (https://www.ncbi.nlm.nih.gov/snp/, accessed on September 5). Origin of genetic ancestry (OGA) is defined within the Allele Frequency Database, ALFRED [25]. The ALFRED database was chosen due to its inclusion of multiple samplings for the same OGA. The average rate in a given OGA (Table 1) was calculated as the weighted average. All calculations and data visualizations were conducted in R.

## Results

### ACE

**Fig. 1:**
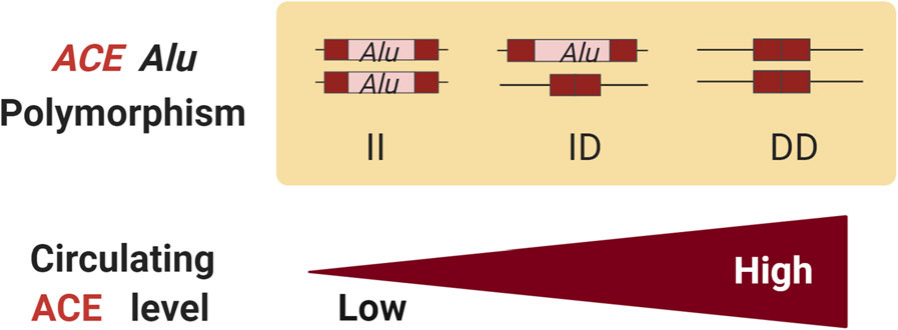
*Alu* polymorphism within *ACE* intron 16 influences circulating ACE enzyme. *ACE* II, ID, and DD are three *ACE* genotypes generated by *Alu* insertion/deletion events. These genotypes affect the circulating level of ACE encoded by the gene. Specifically, individuals with the II genotype have the lowest circulating ACE levels whereas individuals having the DD genotype tend to have the highest-circulating ACE level. I, insertion; D, deletion

ACE (angiotensin-converting enzyme) is a ubiquitously expressed carboxypeptidase and is a central player in the hormonal regulation of the cardiovascular system, namely, the RAAS and the kallikrein-kinin system (KKS) [28]. ACE converts angiotensin I to angiotensin II, which leads to the internalization of ACE2 when in excess [29]. The antagonistic roles that ACE and ACE2 play in inflammation and anti-inflammation are crucial to physiological homeostasis and pathophysiology in the development of hypertension, renal diseases, and ARDS [6, 30–33]. The specific mechanisms implicating the RAAS and the KKS in cytokine storms during COVID-19 have been extensively reviewed [34]. Angiotensin IV, the metabolic product of angiotensin II without the participation of ACE2, modulates endothelial functions, such as plasminogen activator inhibitor-1 (PAI-1) release, which will be discussed further below [35, 36]. ACE acts as kininase to degrade bradykinin, leading to vasoconstriction, further promoting the milieu for thromboinflammation [28]. Interestingly, an intronic *Alu* polymorphism of *ACE* has been shown to directly influence the expression of the gene; individuals having a homozygous absence/deletion of the *Alu* (DD genotype) exhibit the highest expression of ACE, and those with homozygous insertion (II genotype) the lowest [37] (Fig. 1). The polymorphic *Alu* insertion is an AluYa5 element and is in complete linkage disequilibrium (LD) with an intronic variant (rs4341) and a synonymous variant (rs4343); therefore, these SNPs can be used as proxies for genotyping *ACE Alu* I/D (insertion/deletion) [38–41]. At the molecular level, the presence of the *Alu* element within intron 16 has been determined to influence the promoter activity of *ACE*, possibly serving as a *trans*-acting repressor of RNA polymerase II activity [42, 43].

A critical observation is that the *Alu* polymorphism in *ACE* may influence the susceptibility, clinical manifestations, and outcome of COVID-19 infection multidimensionally. First, ACE can downregulate ACE2; individuals with the DD genotype would be predicted to have lower ACE2 expression, therefore, less susceptible to infection. This theory is epidemiologically supported by a negative correlation between the prevalence of COVID-19 and the *ACE* D-allele frequency [19]. As would be predicted, individuals of *ACE Alu* DD genotype have a higher propensity for developing ARDS in respiratory infections, such as Influenza A [44, 45]. Furthermore, the *ACE Alu* I/D polymorphism has been extensively studied across the world for a wide range of cardiovascular diseases (CVDs) and, in light of the available data, we propose closer attention paid to the *ACE Alu* polymorphism when considering the high prevalence of cardiovascular complications observed in COVID-19 [24]. Several studies pointed to a bleeding tendency in individuals with the II genotype, potentially through less robust coagulation, and/or enhanced fibrinolysis [46–48]. Studies characterizing the effects of the *Alu* I/D on the outcome of infectious diseases and CVDs since 2015 are provided in Supplementary Table 1. Interestingly, among patients with myocardial infarction (MI), the D-allele of *ACE* is associated with higher levels of IL-6, which has been associated with worse outcomes of COVID-19 [1, 49]. Considering studies of the *ACE* I/D polymorphism in human population genetics, the variant may in part underlie the biological factors contributing to individual race/ethnicity-related host response observed in the clinical manifestations and outcomes of COVID-19 [50].

### PGR

Progesterone receptors (PGRs) are intracellular receptors that translocate to the nucleus to regulate gene expression through interacting with hormone response elements (HRE) after binding to progesterone or its derivatives [51]. Besides its well-recognized role in obstetrics and gynecology, progesterone has been studied as a potent immunomodulator in regulating many aspects of the innate and adaptive immune response [52, 53]. The relevance of progesterone with respect to vascular system function is also well-recognized. Of interest, arteries have the highest expression of *PGR* besides components of the female reproductive system (https://www.gtexportal.org/home/gene/ENSG00000082175). Sex-specific upregulation of endothelial mineralocorticoid receptors by endothelial progesterone receptor activation occurs in female animals with menstrual cycles [54].

**Fig. 2:**
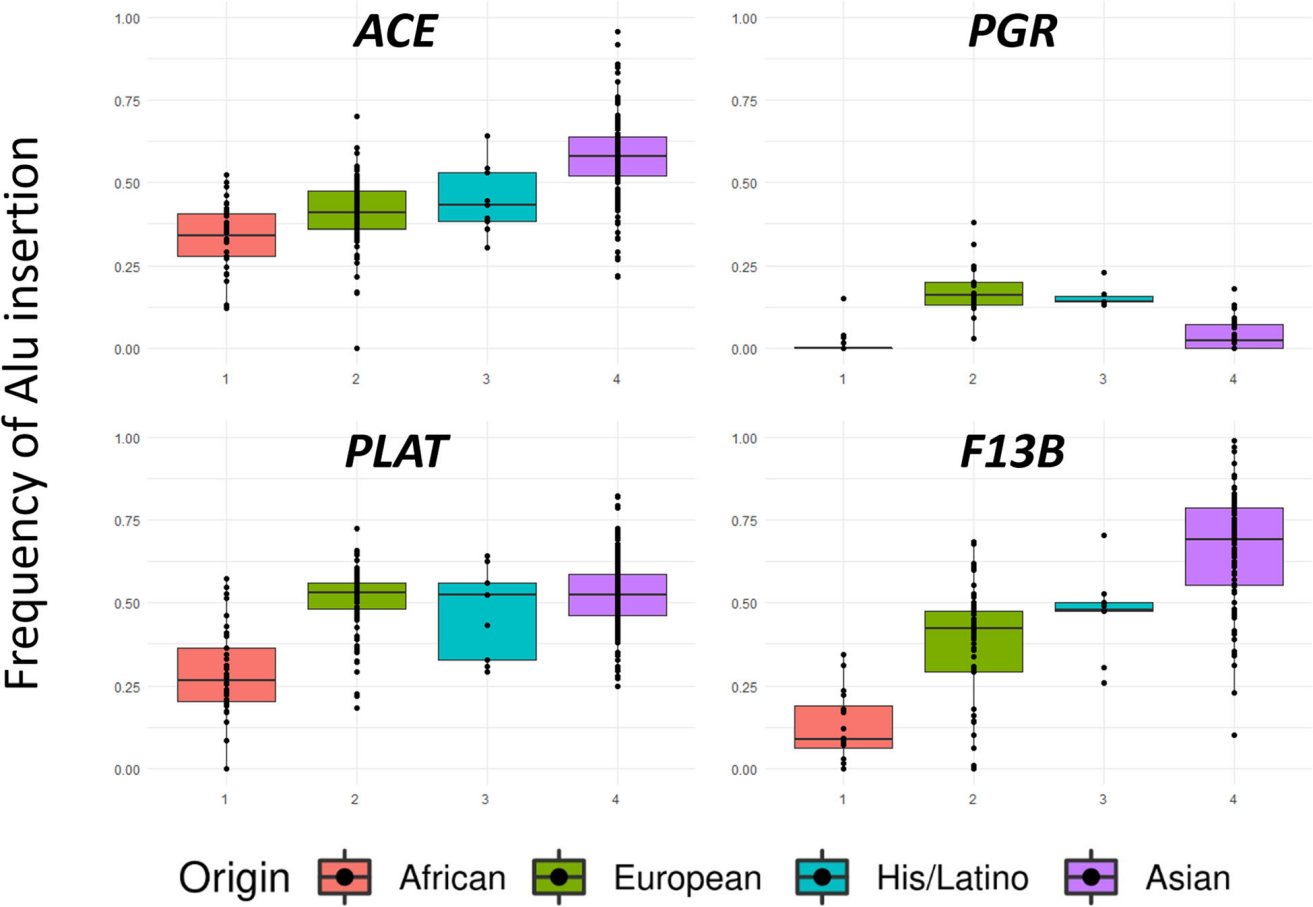
The frequency of polymorphic *Alu* insertion in populations of different origin of genetic ancestry (OGA). OGAs are arranged to show an ascending frequency of *Alu* insertion in *ACE* for visualization purposes*. ACE* and *F13B* are similarly correlated with OGA, where it is the lowest in individuals with genetic ancestry associated with African populations and the highest in Asian populations. The *Alu* polymorphism in *PLAT* shares the trend with *ACE* and *F13B* but not as closely. The frequency of *Alu* insertion in *PGR* does not assume the increase observed with the polymorphisms in the other three genes. We recognize that genetic data have been misused to stigmatize ethnic groups or individuals and the usage of *Alu* frequencies reported herein for such purposes will not be tolerated ethically and is not scientifically justified

Intron 7 of the *PGR* gene has a polymorphic AluYa5 insertion that is in complete LD with two SNPs affecting exons—V660L in exon 4 and H770H in exon 5; these polymorphisms are collectively known as PROGINS [55]. Sex steroid hormones and their downstream signaling have been hypothesized to contribute to the observed difference since they have different regulatory roles in the host immune response to COVID-19 infection [56]. Accordingly, at least one clinical trial has launched within the USA that focuses on the administration of progesterone to male patients having severe cases of COVID-19, as an attempt to mitigate mortality (ClinicalTrials.gov). In light of previous studies, we hypothesize that the *Alu*-related PROGINS contribute to differential host immune response. In particular, Romano and colleagues have shown that the intronic insertion of *Alu* decreases the responsiveness of the receptor to progestin due to a less stable transcript and proteins [57]. From the perspective of infectious diseases, *PGR Alu* I/D has only been investigated in the context of hepatitis E virus (HEV) and human immunodeficiency virus (HIV) [58, 59]. Regarding critical pathways related to SARS-CoV-2 infection, progesterone signaling can upregulate ACE2 in endothelial cells, thus implicating the differential response to progesterone via *PGR Alu* I/D in the clinical outcome of COVID-19 as it shares an intricate connection to the RAAS system [60, 61]. However, sex-related effects conferred by *PGR Alu* I/D remain unclear. Higher levels of progesterone in females may underly key functional differences with respect to males, and there are documented sex-specific differences in the RAAS, KKS, and fibrinolysis being affected by polymorphisms similar to those discussed herein [62]. The frequency of PROGINS does not share the trend of genetic ancestry with the *Alu* polymorphisms in *ACE*, *F13B*, and *PLAT* (Fig. 2). Nonetheless, dual characterization of *Alu* PROGINS and COVID-19 clinical manifestation might offer important insights into the observed ethnic and sex differences underlying the host response to SARS-CoV-2 infection.

### PLAT

*PLAT* encodes for tissue plasminogen activator (tPA), a serine protease found on endothelial cells that promotes fibrinolysis. It functions by converting plasminogen into plasmin, which degrades fibrin polymers, the blood clots, into D-dimers among other degradation products. The activity of tPA is inhibited by PAIs mainly secreted by endothelial cells, among other cell types and tissues. tPA is currently given as a treatment for various CVDs and ARDS in COVID-19 [63, 64], whereas elevated PAI-1 is a risk factor for both thromboembolism and atherosclerosis [65]. Both *PLAT* and *SERPINE1* have polymorphic loci among individuals and have been studied either together or independently for predispositions and prevalence of CVDs [66]. Interestingly, levels of tPA and PAI-1 are epistatically affected by polymorphisms in the RAAS, KKS, and the fibrinolysis system [67].

Here, we focus on the structural variation caused by a polymorphic AluYa5 insertion in intron 8 of *PLAT* [68, 69]. The molecular effects and mechanisms have not been elucidated for this polymorphic *Alu* insertion; however, it was observed that individuals of the *Alu* II genotype have a higher “in vivo” release rate of tPA from vascular endothelial cells when compared to those with other *PLAT Alu* I/D genotypes [70]. Moreover, bacterial osteomyelitis patients with the *PLAT Alu* II genotype had lower plasma PAI-1/tPA complex levels and likely more circulating tPA [71]. This is not only very interesting in the context of bacterial infection but also potentially important for SARS-CoV-2 infection—although more active tPA mitigates the risk for developing complications of thromboembolism nature, insufficient coagulation could compromise the body’s ability to limit the pathogen locally, predisposing the individual to viremia of higher viral load and likely more severe systemic infection. In light of previous research, the *PLAT Alu* polymorphism, with regards to the current COVID-19 pandemic, could provide clues to the differential occurrence of coagulation and fibrinolysis dysfunction. If the effect of the *Alu* polymorphism was as predicted by previous studies, we expect to see individuals with the *PLAT Alu* DD genotype at a higher risk for developing diseases of hypercoagulative nature, such as pulmonary embolism (PE), venous thromboembolism, myocardial infarction, and stroke. In contrast, *PLAT Alu* II individuals would be expected to have increased risk of a variety of organ damage. It is worth noting that Ang IV (angiotensin IV) synthesis, when ACE/ACE2 ratio is higher, in the RAAS regulates the release of PAI-1 from endothelial cells; this provides the basis for potential interaction between *ACE Alu* I/D and *PLAT Alu* I/D polymorphisms [35]. Interestingly, the thrombotic syndrome in COVID-19 is similar to complement-mediated thrombotic microangiopathy (TMA) [72]. As plasminogen and plasmin can serve as a complement inhibitor and C5 convertase, respectively, the *PLAT Alu* I/D could also affect complement activation in COVID-19 [73]. Given the *PLAT Alu* I/D polymorphisms are correlated with genetic ancestry (Fig. 2); this variation may offer some biological basis for the observed patient-specific host response to SARS-CoV-2.

### F13B

Plasma factor XIII is a protein in the blood coagulation cascade that stabilizes blood clots by linking fibrin monomers to fibrin polymers. FXIII is a heterotetrameric (A2B2) transglutaminase that contains two different subunits encoded by FXIIIA and FXIIIB. The A subunit is catalytically active while the B subunit protects the A subunit from degradation in circulation. Intron 10 of *F13B* has a polymorphic AluYa5 insertion that has been widely studied in population genetics as a genetic marker as the I-allele frequency is highly correlated with distinct population groups across the world (Fig. 2) [38, 39, 74, 75].

FXIII functions antagonistically with tPA in the blood coagulation cascade; therefore, the *Alu* polymorphism may prove to be essential for better prediction of the potential complications an individual might develop during COVID-19 infection for similar reasons described above. FXIIIB has been associated with the development of various bleeding disorders in recent years, including ischemic stroke (IS), venous thrombosis (VTE), coronary artery disease, and MI [76–78]. Of interest, the polymorphic AluYa5 insertion in *F13B* has been studied for conferring the risk of coronary atherosclerosis [79]. A C-to-G polymorphism in intron 11 can result in allele-specific alternative splicing that correlates with decreased FXIII albeit the significance of its level is controversial [76, 78, 80–82]. While it is not linked to any known SNPs, the AluYa5 insertion in intron 10 could be in LD with the C-to-G polymorphism in intron 11 given the two variants have similar racial/ethnic attributes among human populations [80]. Additional studies focused on the polymorphic *Alu* may elucidate additional molecular mechanisms underlying the functional regulation of FXIII. Further, the interaction between FXIIIB and proteins in the complement system, such as C3 and C1q, deserves attention for COVID-19 as reasoned in the previous section [83]. Similar to the *ACE* and *PLAT Alu* I/D polymorphism, the *F13B* variation observed across the human population could be an important biological factor when considering the variable individual response to COVID-19.

## Discussion

SARS-CoV-2 is spreading rapidly across the globe, readily causing both pulmonary and extrapulmonary symptoms across the human population. Clinical presentations and outcomes of COVID-19 are highly variable and have been linked to race as well as sex. We reviewed studies surrounding polymorphic *Alu* retrotransposons in *ACE*, *PGR*, *PLAT*, and *F13B* that have been either mechanistically linked to and/or associated with clinical conditions relevant to COVID-19. In light of these studies, we posit that primate-specific *Alu* retrotransposons are actively influencing host physiology with respect to SARS-CoV-2 infection and are contributing to the diverse spectrum of observed COVID-19 symptoms. Moreover, variable populational rates of *Alu* insertion in the genes highlighted herein may provide novel insights into understanding the biological basis for the racial/ethnic discrepancies observed in the outcome of COVID-19.

**Fig. 3:**
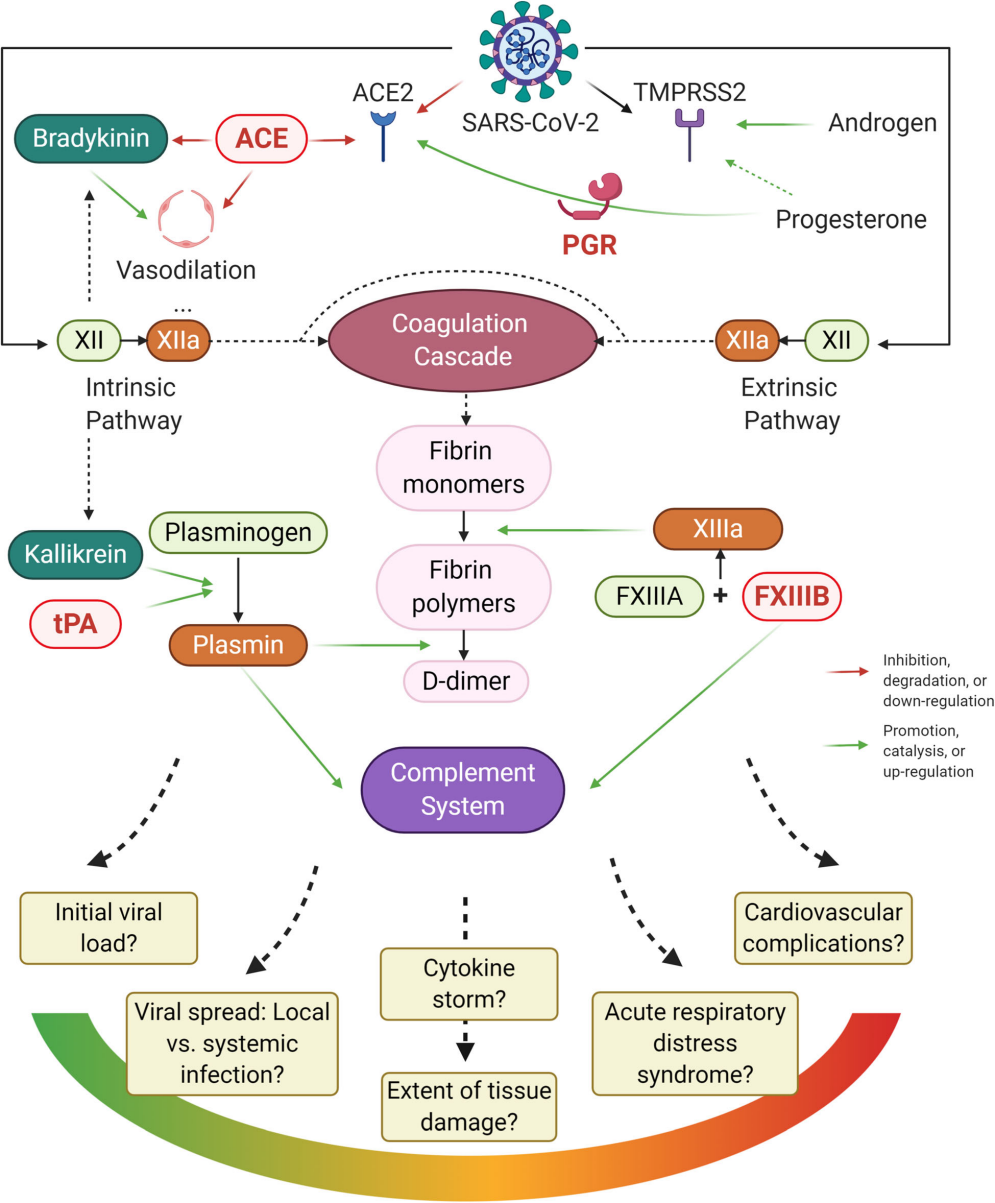
SARS-CoV-2 and relevant biological processes that involve ACE, PGR, FXIIIB, and tPA. SARS-CoV-2 uses ACE2 as a receptor for cell entry, facilitated by androgen-regulated TMPRSS2. SARS-CoV-2 can cause tissue and endothelial cell damages, activating the intertwined intrinsic and extrinsic blood coagulation pathways. ACE and ACE2 are two essential regulatory components in the RAAS. ACE can promote vasoconstriction through antagonizing the regulatory role of ACE2 in the RAAS and acting as a bradykininase in the KKS additionally activated by the intrinsic coagulation pathway. ACE2 is locally downregulated by SARS-CoV-2 entry and excessive ACE signaling. Progesterone can upregulate ACE2 via binding to the intracellular progesterone receptor. The result of hemostasis is the conversion of fibrin monomers to polymers catalyzed by Factor XIII, which, in circulation, is a heterotetrameric protein composed of FXIIIA and FXIIIB subunits. D-dimers constitute one of the fibrinolysis degradation products generated as plasmin catalyzes fibrinolysis. Plasminogen is the zymogen of plasmin; the transition can be facilitated by kallikrein and tPA among other molecules. Both plasmin and FXIIIB can activate the complement system in addition to their function as catalysts. The functions of proteins encoded by the *ACE*, *PGR*, *PLAT*, and *F13B* are integrally essential to the RAAS, KKS, and fibrinolytic systems; *Alu* variants in these genes likely contribute to the spectrum of symptoms observed in COVID-19 cases. ACE, angiotensin-converting enzyme; FXIII, Factor XIII; KKS, kallikrein-kinin system; PGR, progesterone receptor; RAAS, renin-angiotensin-aldosterone system; SARS-CoV-2, severe acute respiratory syndrome coronavirus 2; TMPRSS2, transmembrane protease, serine 2; tPA, tissue plasminogen activator

The *Alu* polymorphisms that we reviewed here in *ACE*, *PGR*, *PLAT*, and *F13B* constitute an example of potentially important alleles (or a combination of) that would not be identified by GWAS and non-targeted sequencing techniques. Firstly, GWAS has been mostly unsuccessful in detecting epistatic effects in humans [10, 13]. Polymorphisms in genes from the RAAS, KKS, and the fibrinolytic system, including *ACE Alu* I/D, are known to exert epistatic effects on the development of cardiovascular accidents [67]. When considering the available data, the *Alu* polymorphisms in *PGR*, *PLAT*, and *F13B* in addition to *ACE* are likely epistatically important to the homeostasis of inflammation and vascular systems and would potentially be undetected by a GWAS approach (Fig. 3). *Alu* polymorphisms in *ACE* and *PGR* have potential to influence the susceptibility of individuals and influence the initial viral load and tissue damage by SARS-CoV-2 by affecting the level of circulating ACE and PGR. Downregulation of ACE2 upon cell entry of SARS-CoV-2 impacts the physiological balance between ACE and ACE2, which are two essential regulatory components in the RAAS [28]. We posit that individuals with the *ACE Alu* DD genotype may be predisposed to complications of COVID-19 due to higher baseline ACE levels. Patients of advanced age and cardiovascular diseases or diabetes have alterations in ACE expression and Ang II signaling [84–86], likely rendering them more susceptible to further repercussions of ACE/ACE2 imbalance. Progesterone can upregulate ACE2 likely via transcription regulatory effects of the nuclear receptor PGR [61]. Moreover, the sensitivity to progesterone, the level of ACE, and the functions of FXIII and tPA affected by *Alu* structural variations can influence the spread of SARS-CoC-2 in circulation as well as predispose individuals to different tendencies of developing cardiovascular conditions (Fig. 3). SARS-CoV-2 can cause tissue and endothelial cell damage, activating the intertwined intrinsic and extrinsic blood coagulation pathways [1]. Progesterone is known to have anti-inflammatory and vasoactive effects [56, 87]. The intrinsic coagulation pathway can activate the bradykininase activity of ACE in the KKS, further promoting vasoconstriction and production of cytokines, including IL-6 [88, 89] (Fig. 3). Hemostasis is achieved through the conversion of fibrin monomers to polymers catalyzed by Factor XIII composed of FXIIIA and FXIIIB subunits in circulation. tPA and kallikrein, among other molecules, catalyze the conversion of plasminogen to plasmin, which in turn catalyzes fibrinolysis, producing D-dimer and other fibrinolysis degradation products (Fig. 3). Further, both plasmin and FXIIIB can activate the complement system in addition to their function in the fibrinolysis system [90] (Fig. 3). Dysregulated coagulation and fibrinolysis, exemplarily manifested as pulmonary embolism, cardiovascular accidents, and acute respiratory distress syndrome, are common pathophysiology observed for severe COVID-19 cases.

Given the biological complexity of the human body, it is unlikely that a small number of SNPs in a handful of genes are independently responsible for the differential host response among the human population across the world. There are over one million copies of *Alu*s located in the human genome and many of these mobile elements exhibit lineage-specific mobilization and epigenetic patterns that are linked to human demographics and individual natural history traits. The *Alu* polymorphisms presented here represent a small fraction of an individual’s global retrotransposon population, however, they likely exert epistatic efforts with other *Alu* and/or known non-*Alu* polymorphisms in the discussed systems [67]. Genetic and epigenetic mosaicisms of non-polymorphic *Alus* as well as polymorphic *Alu*s located in other genes, particularly the apolipoprotein cluster (*APOA1*, *APOC3*, *APOA4*) and *HLA* loci [91–93], could also influence the host response and disease outcomes of COVID-19. Enhanced transcription of *Alus* during viral infection is a known phenomenon and the expression of *Alu* and other retrotransposons in human cells have been found to increase during coronavirus infection [94]. Double-stranded RNAs transcribed from *Alu* elements have been shown to activate antiviral innate immune signaling pathways in mitochondria through MDA5 [95]. Future efforts in characterizing the polymorphism as well as genetic and epigenetic mosaicisms of retrotransposons could reveal their physiological and pathological roles in modulating inflammation and the immune response. Such knowledge may improve our understanding of both cellular and systemic host responses to viral infections beyond COVID-19.

## Conclusions

COVID-19 is a highly infectious respiratory disease caused by SARS-CoV-2, affecting multiple organs and systems in infected individuals through a complicated web of factors. In the current pandemic, clinical manifestations and outcomes of COVID-19 diverge with sex as well as differences in genetic ancestry and/or socioeconomic inequalities. Here, we present a prospective paradigm for Alu elements that has the potential to help explain differential host response to SARS-CoV-2 infection. *Alu* retrotransposons have been extensively researched from the perspective of human evolution and population genetics. They are well known to influence human health and disease outcomes, including those associated with genetic ancestry. Indeed, an *Alu* variant within the *ACE* gene has already been hypothesized to impact COVID-19 susceptibility and morbidity and, in light of this, several *Alu* variants are likely influencing host response pathways. To this end, we highlighted *Alu* polymorphisms in four genes encoding for ACE, PGR, tPA, and FXIIIB that are integral components of the RAAS, KKS, endocrine system, coagulation cascade, fibrinolysis, and the complement system (Fig. 3). These systems and processes are highly relevant to the development of major clinical complications of SARS-CoV-2 infection, including IL-6 levels, elevated D-dimer and fibrinogen, MI, IS, VTE, PE, and the cytokine storm as reviewed by Gupta and colleagues [1]. We posit that these *Alu* variants and/or the potential epistatic effects influence the clinical presentations of and host immune response to SARS-CoV-2 infection. Moreover, recently discovered epigenetic gene regulatory pathways that center on *Alu* elements might help explain the heterogeneity of symptoms observed across COVID-19 patients. If accurate, additional research focused on these *Alu*-related mechanisms could yield novel genetic markers capable of predicting the clinical outcomes as well as patient-specific treatment strategies for COVID-19.

## Supplementary Information

**Additional file 1: Supplementary Table 1.** Studies characterizing the effect that the *Alu* I/D within intron 16 of the ACE gene has on the outcome of various cardiovascular and infectious diseases. See Fig. 1 for depiction of the *Alu* polymorphism. Note that several studies highlighted within the table are underpowered and we recommend additional research having greater sample sizes.

## Data Availability

All data generated or analyzed during this study are included in this published article and its supplementary information files.

## Abbreviations

ACE: Angiotensin-converting enzyme
ACE2: Angiotensin-converting enzyme 2
Ang IV: Angiotensin IV
ARDS: Acute respiratory distress syndrome
COVID-19: Coronavirus disease 2019
CVDs: Cardiovascular diseases
GWAS: Genome-wide association study
HEV: Hepatitis E virus
HIV: Human immunodeficiency virus
HRE: Hormone response elements
I/D: Insertion/deletion
IL-6: Interleukin-6
IS: Ischemic stroke
KKS: Kallikrein-kinin system
LD: Linkage disequilibrium
MI: Myocardial infarction
PAI-1: Plasminogen activator inhibitor-1
PE: Pulmonary embolism
PGR: Progesterone receptor
RAAS: Renin-angiotensin-aldosterone system
SARS-CoV-2: Severe acute respiratory syndrome coronavirus 2
SNP: Single-nucleotide polymorphism
TMPRSS2: Transmembrane Serine Protease 2
tPA: Tissue plasminogen activator
VTE: Venous thrombosis
