# Supplementary material for: Alu retrotransposons and COVID-19 susceptibility and morbidity
Source: Hum Genomics. 2021 Jan 4;15:2. doi: 10.1186/s40246-020-00299-9 (PMC7779329; doi:10.1186/s40246-020-00299-9)
Supplement: Supplementary file 1 — Additional file 1: Supplementary Table 1. Studies characterizing the effect that the Alu I/D within intron 16 of the ACE gene has on the outcome of various cardiovascular and infectious diseases. See Fig. 1 for depiction of the Alu polymorphism. Note that several studies highlighted within the table are underpowered and we recommend additional research having greater sample sizes. [file 40246_2020_299_MOESM1_ESM.docx]

**Supplementary Table 1.** Studies characterizing the effect that the *Alu* I/D within intron 16 of the ACE gene has on the outcome of various cardiovascular and infectious diseases. See Figure 1 for depiction of the *Alu* polymorphism. Note that several studies highlighted within the table are underpowered and we recommend additional research having greater sample sizes.

| **Infectious diseases** | **Year** | **Sample** | **Control** | **Region/**  **Nationality/**  **Race** | **Findings regarding ACE I/D polymorphism** | **Ref.** |
| --- | --- | --- | --- | --- | --- | --- |
| **COVID-19** | 2020 | Meta-analysis | | Multiple | II genotype frequency was negatively correlated with SARS-CoV-2 infection and mortality rate | [1] |
|  | 2020 | Meta-analysis | | Multiple | Increased ratio of I/D allele frequency was positively correlated with the recovery rate and negatively with the mortality rate | [2] |
|  | 2020 | 137 non-severe  67 severe-ICU | 536^a^ | Spain | DD genotype was associated with a worse outcome of COVID-19 | [3] |
|  | 2020 | Meta-analysis | | Asia | The D allele frequency was positively correlated with SARS-CoV-2 infection and mortality rate | [4] |
|  | 2020 | Meta-analysis | | Multiple | Higher AD allele frequency was negatively correlated with the infection rate | [5] |
| **Community-acquired pneumonia (CAP)** | 2020 | 300 patients with CAP | 300 | Egypt | Frequencies of DD genotype and D allele were overrepresented in CAP patients | [6] |
| **Chagas heart disease** | 2020 | 343 patients with positive serology for Chagas disease (CD) at different stages | | Brazil | DD genotype individuals and D carriers were overrepresented in CD patients with heart failure | [7] |
| **Kawasaki disease** | 2017 | Meta-analysis | | Asia | Inconsistent findings when different statistical adjustments were applied | [8] |
| **Acute respiratory distress syndrome** | 2015 | Meta-analysis | | Asians and Caucasians | 1) DD genotype was associated with increased risk of ARDS in Caucasians but not Asians 2) ARDS was more common in adults | [9] |

| **Cardiovascular disorders/**  **functions** | **Year** | **Sample** | **Control** | **Region/**  **Nationality/**  **Race** | **Results** | **Ref.** |
| --- | --- | --- | --- | --- | --- | --- |
| **Aortic dissection and aneurysm** | 2018 | 38 patients with aortic dissections 67 patients with aortic aneurysms | 60 | Turkey | A higher rate of ID genotype was found in the aneurysm group and DD genotype in the dissection group | [10] |
| **Aneurysmal subarachnoid hemorrhage (aSAH)** | 2017 | 149 aSAH patients | 50 | United States | No association was found between I/D polymorphism and the development of aSAH | [11] |
| **Abdominal aortic aneurysm (AAA)** | 2016 | 117 | 117 | Croatia | Allele D and DD genotype were more prevalent in AAA patients | [12] |
| **Aortic Aneurysm (AA)** | 2015 | Meta-analysis | | Multiple | In Caucasians but not Asians, DD genotype was significantly associated with developing AA, specifically AAA but not thoracic AA | [13] |
| **Atherosclerosis (AS)** | 2019 | Meta-analysis | | Multiple | 1) The D allele was associated with the risk of AS, especially in Europeans but not Asians 2) Copy number of D allele was positively associated with the AS risk in a dose-dependent manner | [14] |
|  | 2019 | 154 | 145 | Iran | No association was found for ACE I/D polymorphism and the risk of AS. | [15] |
| **Atrial fibrillation** | 2015 | Meta-analysis | | Multiple | DD genotype was associated with a higher risk of developing AF | [16] |
| **Premature coronary artery atherosclerosis** | 2020 | 407 participants (no older than 55) with premature coronary artery disease (CAD) | | Poland | DD genotype was associated with higher plasma fibrinogen levels female but not male patients | [17] |
| **Coronary artery atherosclerosis** | 2018 | 145 | 300 | Tunisian | D allele frequency and DD genotype were significantly more prevalent among patients with CAD | [18] |
|  | 2017 | 330 | 332 | Asian Indians | Frequencies of DD genotype and D allele were statistically higher in CAD patients | [19] |
| **Coronary artery disease** | 2017 | 187 | 185 | South Indians | DD genotype was a risk factor for CAD patients with type 2 diabetes mellitus (T2DM) | [20] |
| **Carotid atherosclerosis with T2DM** | 2016 | 595 | 200 | Slovenian | T2DM patients of DD genotype had a faster progression of AS | [21] |
| **Coronary artery disease** | 2016 | 171 | 123 | Bulgarian | D allele carriers were more likely to develop the acute coronary syndrome | [22] |
| **Coronary heart disease (CHD)** | 2015 | 161 | 1954 | Japan | DD genotype significantly accelerated the development of CHD | [23] |
| **Coronary restenosis** | 2015 | Meta-analysis | | Multiple | D allele carriers were at risk for developing coronary restenosis in subjects < 60 years old in both Caucasian and Asian populations | [24] |
| **Congenital heart disease** | 2015 | 96 | 145 | Saudi | No association was found between the occurrence of congenital heart disease and the ACE I/D polymorphism | [25] |
| **Diastolic heart failure** | 2016 | 176 | 88 | Iran | D allele was significantly more prevalent among patients with LVH when compared to controls and those without LVH | [26] |
| **Left Ventricular Hypertrophy (LVH)** | 2019 | Meta-analysis | | India | D allele was associated with increased risk of LVH | [27] |
| **Hypertrophic cardiomyopathy  (HCM)** | 2017 | Meta-analysis | | Multiple | D allele frequency was more prevalent in HCM patients | [28] |
|  | 2016 | 168 | 136 | Spain | No association was found between ACE I/D polymorphism and the development of HCM | [29] |
|  | 2015 | 155 non-diabetic hypertensive individuals | | Brazil | 1) DD genotype was more prevalent in ventricular hypertrophy (LVH) patients 2) Carriers of the D allele had a higher systolic BP during wakefulness, left ventricular mass, and higher prevalence of LVH | [30] |
| **Peripartum cardiomyopathy (PPCM)** | 2018 | 45 | 70 | India | 1) DD genotype and D allele frequency was higher in PPCM patients 2) DD genotype was associated with worse individual systolic performance indices through echocardiography | [31] |
| **Ischemic stroke (IS)** | 2020 | 70 | 30 | Iran | Higher D allele frequency was observed in IS patients, but the difference was not statistically significant | [32] |
|  | 2019 | 94 IS patients treated with rt-PA | | Serbia | Patients of ACE II genotype more readily developed hemorrhagic transformation after treatment | [33] |
|  | 2017 | Meta-analysis | | Multiple | Patients with DD genotype were at higher risks of developing IS | [34] |
|  | 2016 | 35 with perinatal arterial IS 38 with childhood arterial IS | 100^b^ | Croatia | No association was found between ACE I/D polymorphism and the risk of developing IS | [35] |
|  | 2016 | Meta-analysis | | Caucasian | DD genotype was associated with the increased risk of IS in Caucasians | [36] |
|  | 2016 | 60 | 30 | Egyptian | DD genotype was statistically more prevalent in patients with IS | [37] |
| **Ischemic and hemorrhagic stroke (IS/HS)** | 2015 | 200 IS patients  200 HS patients | 200 | India | 1) DD genotype and D allele were risk factors for the development of IS and HS 2) DD genotype individuals were more likely to develop IS over HS  3) Mean ACE activity level was significantly higher in IS and HS but similar between the two | [38] |
| **Myocardial infarction (MI)** | 2019 | 199 | 216 | China | DD genotype and D allele were positively correlated with ACE, kallikrein, and IL-6 levels but not with angiotensin II | [39] |
|  | 2017 | 140 | 182 | Morocco | 1) D allele frequency was higher in MI patients  2) DD genotype and D allele were more prevalent in patients less than 45 years old than those that are over. | [40] |
|  | 2016 | 108 patients with acute MI | | Turkey | D allele frequency carriers had a higher magnitude of the QT dispersion at both baseline and the end of the six-month follow-up | [41] |
|  | 2016 | 200 | 200^b^ | India | II genotype was a risk factor for developing MI, especially in individuals with low cardiovascular risk | [42] |
|  | 2015 | Meta-analysis | | Han Chinese | D allele was a risk factor for MI in the Chinese Han population | [43] |
| **Refractory hyperaldosteronism in heart failure** | 2015 | 109 patients with left ventricular systolic dysfunction (LVSD) | | Italy | No significant association was found between ACE I/D polymorphism and refractory hyperaldosteronism in heart failure | [44] |
| **Venous thromboembolism (VTE)** | 2018 | 310 | 289^b^ | Turkey | No association was found between ACE I/D polymorphism and VTE | [45] |
| **Deep vein thromboembolism (DVT)** | 2016 | 193 | 500 | Iran | No association was found between ACE I/D polymorphism and DVT | [46] |
| **Peripheral vascular disease (PVD)** | 2017 | Meta-analysis | | Multiple | DD genotype and D allele were statistically associated with PVD susceptibility in Asians but not in Caucasians | [47] |
| **Thrombosis** | 2015 | 104 | 106 | Brazil | No association was found between ACE I/D polymorphism and thrombosis | [48] |
| **Intracranial Hemorrhage (ICH)** | 2020 | Meta-analysis | | Multiple | DD genotype and D allele were risk factors for the development of ICH | [49] |
| **Myocardial perfusion** | 2019 | 810 with known or suspected CAD | | Greece | Among the polymorphisms investigated, the D allele had the strongest association with abnormal myocardial perfusion. | [50] |

a. age-matched

b. age, gender-matched

**References:**

1. Yamamoto N, Ariumi Y, Nishida N, Yamamoto R, Bauer G, Gojobori T, et al. SARS-CoV-2 infections and COVID-19 mortalities strongly correlate with ACE1 I/D genotype. Gene. 2020;758:144944.

2. Hatami N, Ahi S, Sadeghinikoo A, Foroughian M, Javdani F, Kalani N, et al. Worldwide ACE (I/D) polymorphism may affect COVID-19 recovery rate: An ecological meta-regression. Endocrine. 2020;68:479–84.

3. Gómez J, Albaiceta GM, García-Clemente M, López-Larrea C, Amado-Rodríguez L, Lopez-Alonso I, et al. Angiotensin-converting enzymes (ACE, ACE2) gene variants and COVID-19 outcome. Gene. 2020;762:145102.

4. Pati A, Mahto H, Padhi S, Panda AK. ACE deletion allele is associated with susceptibility to SARS-CoV-2 infection and mortality rate: An epidemiological study in the Asian population. Clin Chim Acta. 2020;510:455–8.

5. Delanghe JR, Speeckaert MM, Buyzere ML De. The host’s angiotensin-converting enzyme polymorphism may explain epidemiological findings in COVID-19 infections. Clin Chim Acta. 2020;505:192–3.

6. Abouzeid H, Alkholy UM, Abdou MA, Morsy SM, Abdelrahman HM, Sherif AM, et al. Angiotensin-converting enzyme insertion/deletion gene polymorphism in Egyptian children with CAP: A case-control study. Pediatr Pulmonol. 2017;52:1592–8.

7. Alves SMM, Alvarado-ArnÃ\textordfemenines LE, Cavalcanti M da GA de M, Carrazzone C de FV, Pacheco AGF, Sarteschi C, et al. Influence of angiotensin-converting enzyme insertion/deletion gene polymorphism in progression of Chagas heart disease. Rev Soc Bras Med Trop. 2020;53.

8. Pan Y, Lu H, Lu H. Angiotensin-converting enzyme insertion/deletion polymorphism and susceptibility to Kawasaki disease: A meta-analysis . African Health Sciences. 2017;17:991–9.

9. Deng X, Zhang S, Jin K, Li L, Gu W, Liu M, et al. Angiotensin-converting enzyme I/D polymorphism and acute respiratory distress syndrome. J Renin Angiotensin Aldosterone Syst. 2015;16:780–6.

10. Özmen R, Tunçay A, Şener EF, Emiroğulları ÖN. Relationship of genetic factors with development of aortic dissection and aneurysm. Turk gogus kalp damar cerrahisi Derg. 2018;26:557–64.

11. Griessenauer CJ, Tubbs RS, Foreman PM, Chua MH, Vyas NA, Lipsky RH, et al. Association of renin-angiotensin system genetic polymorphisms and aneurysmal subarachnoid hemorrhage. J Neurosurg. 2018;128:86–93.

12. Crkvenac Gregorek A, Gornik KC, Polancec DS, Dabelic S. Association of 1166A>C AT(1)R, -1562C>T MMP-9, ACE I/D, and CCR5Δ32 polymorphisms with abdominal aortic aneurysm in Croatian patients. Genet Test Mol Biomarkers. 2016;20:616–23.

13. Song Y, Miao R, Wang H, Qin X, Zhang Y, Miao C, et al. Meta-analysis of the association between angiotensin-converting enzyme I/D polymorphism and aortic aneurysm risk. J Renin Angiotensin Aldosterone Syst. 2015;16:1125–9.

14. Xia M, Wang M, Jiang H, Liu Y, Ma L, Lu C, et al. Association of angiotensin-converting enzyme insertion/deletion polymorphism with the risk of atherosclerosis. J Stroke Cerebrovasc Dis. 2019;28:1732–43.

15. Nouryazdan N, Adibhesami G, Birjandi M, Heydari R, Yalameha B, Shahsavari G. Study of angiotensin-converting enzyme insertion/deletion polymorphism, enzyme activity and oxidized low density lipoprotein in Western Iranians with atherosclerosis: A case-control study. BMC Cardiovasc Disord. 2019;19:184.

16. Ma R, Li X, Su G, Hong Y, Wu X, Wang J, et al. Angiotensin-converting enzyme insertion/deletion gene polymorphisms associated with risk of atrial fibrillation: A meta-analysis of 23 case-control studies. J Renin Angiotensin Aldosterone Syst. 2015;16:793–800.

17. Kryczka KE, Płoski R, Księżycka E, Kruk M, Kostrzewa G, Kowalik I, et al. The association between the insertion/deletion polymorphism of the angiotensin-converting enzyme gene and the plasma fibrinogen level in women and men with premature coronary artery atherosclerosis. Pol Arch Intern Med. 2020;

18. Amara A, Mrad M, Sayeh A, Lahideb D, Layouni S, Haggui A, et al. The effect of ACE I/D polymorphisms alone and with concomitant risk factors on coronary artery disease. Clin Appl Thromb Hemost. 2018;24:157–63.

19. Bhatti GK, Bhatti JS, Vijayvergiya R, Singh B. Implications of ACE (I/D) gene variants to the genetic susceptibility of coronary artery disease in Asian Indians. Indian J Clin Biochem. 2017;32:163–70.

20. Mani D, Chinniah R, Ravi P, Swaminathan K, Janarthanan RA, Vijayan M, et al. redisposition of angiotensin-converting enzyme deletion/deletion genotype to coronary artery disease with type 2 diabetes mellitus in South India. Indian J Endocrinol Metab. 2017;21:882–5.

21. Merlo S, Novák J, Tkáčová N, Nikolajević Starčević J, Šantl Letonja M, Makuc J, et al. Association of the ACE rs4646994 and rs4341 polymorphisms with the progression of carotid atherosclerosis in slovenian patients with type 2 diabetes mellitus. Balkan J Med Genet. 2015;18:37–42.

22. Mokretar K, Velinov H, Postadzhiyan A, Apostolova M. Association of polymorphisms in endothelial nitric oxide synthesis and renin-angiotensin-aldosterone system with developing of coronary artery disease in Bulgarian patients. Genet Test Mol Biomarkers. 2016;20:67–73.

23. Kondo H, Ninomiya T, Hata J, Hirakawa Y, Yonemoto K, Arima H, et al. Angiotensin I-converting enzyme gene polymorphism enhances the effect of hypercholesterolemia on the risk of coronary heart disease in a general Japanese population: the hisayama study. J Atheroscler Thromb. 2015;22:390–403.

24. Miao H-W, Gong H. Association of ACE insertion or deletion polymorphisms with the risk of coronary restenosis after percutaneous coronary intervention: A meta-analysis. J Renin Angiotensin Aldosterone Syst. 2015;16:844–50.

25. Alazhary NM. Angiotensin-converting enzyme gene insertion deletion (ACE I/D) polymorphism in Saudi children with congenital heart disease. Eur Rev Med Pharmacol Sci. 2015;19:2026–30.

26. Bahramali E, Rajabi M, Jamshidi J, Mousavi SM, Zarghami M, Manafi A, et al. Association of ACE gene D polymorphism with left ventricular hypertrophy in patients with diastolic heart failure: A case-control study. BMJ Open. 2016;6:e010282.

27. Fajar JK, Pikir BS, Sidarta EP, Berlinda Saka PN, Akbar RR, Heriansyah T. The gene polymorphism of angiotensin-converting enzyme intron deletion and angiotensin-converting enzyme G2350A in patients with left ventricular hypertrophy: A meta-analysis. Indian Heart J. 2019;71:199–206.

28. Yuan Y, Meng L, Zhou Y, Lu N. Genetic polymorphism of angiotensin-converting enzyme and hypertrophic cardiomyopathy risk: A systematic review and meta-analysis. Medicine (Baltimore). 2017;96:e8639.

29. García-Honrubia A, Hernández-Romero D, Orenes-Piñero E, Romero-Aniorte AI, Climent V, García M, et al. Clinical implications of nonsarcomeric gene polymorphisms in hypertrophic cardiomyopathy. Eur J Clin Invest. 2016;46:123–9.

30. Cosenso-Martin LN, Vaz-de-Melo RO, Pereira LR, Cesarino CB, Yugar-Toledo JC, Cipullo JP, et al. Angiotensin-converting enzyme insertion/deletion polymorphism, 24-h blood pressure profile and left ventricular hypertrophy in hypertensive individuals: A cross-sectional study. Eur J Med Res. 2015;20:74.

31. Yaqoob I, Tramboo NA, Bhat IA, Pandith A, Beig JR, Hafeez I, et al. Insertion/deletion polymorphism of ACE gene in females with peripartum cardiomyopathy: A case-control study. Indian Heart J. 2018;70:66–70.

32. Salem GM, Gab-Allah GK. Angiotensin converting enzyme polymorphism and ischemic stroke. Neurosciences (Riyadh). 2020;25:176–81.

33. Dušanović Pjević M, Beslac Bumbaširevic L, Vojvodic L, Grk M, Maksimović N, Damnjanović T, et al. Analysis of the association between polymorphisms within PAI-1 and ACE genes and ischemic stroke outcome after rt-PA therapy. J Pharm Pharm Sci. 2019;22:142–9.

34. Wei LK, Au A, Menon S, Griffiths LR, Kooi CW, Irene L, et al. Polymorphisms of MTHFR, eNOS, ACE, AGT, ApoE, PON1, PDE4D, and ischemic stroke: Meta-analysis. J Stroke Cerebrovasc Dis. 2017;26:2482–93.

35. Coen Herak D, Lenicek Krleza J, Radic Antolic M, Horvat I, Djuranovic V, Zrinski Topic R, et al. Association of polymorphisms in coagulation factor genes and enzymes of homocysteine metabolism with arterial ischemic stroke in children. Clin Appl Thromb Hemost. 2017;23:1042–51.

36. Yuan H, Wang X, Xia Q, Ge P, Wang X, Cao X. Angiotensin converting enzyme (I/D) gene polymorphism contributes to ischemic stroke risk in Caucasian individuals: a meta-analysis based on 22 case-control studies. Int J Neurosci. 2016;126:488–98.

37. Mostafa MA, El-Nabiel LM, Fahmy NA, Aref H, Shreef E, Abd El-Tawab F, et al. ACE gene in Egyptian ischemic stroke patients. J Stroke Cerebrovasc Dis. 2016;25:2167–71.

38. Das S, Roy S, Sharma V, Kaul S, Jyothy A, Munshi A. Association of ACE gene I/D polymorphism and ACE levels with hemorrhagic stroke: Comparison with ischemic stroke. Neurol Sci. 2015;36:137–42.

39. Dai S, Ding M, Liang N, Li Z, Li D, Guan L, et al. Associations of ACE I/D polymorphism with the levels of ACE, kallikrein, angiotensin II and interleukin-6 in STEMI patients. Sci Rep. 2019;9:19719.

40. Hmimech W, Idrissi HH, Diakite B, Korchi F, Baghdadi D, Tahri H, et al. Impact of I/D polymorphism of angiotensin-converting enzyme (ACE) gene on myocardial infarction susceptibility among young Moroccan patients. BMC Res Notes. 2017;10:1–6.

41. Karahan Z, Altıntaş B, Uğurlu M, Kaya İ, Uçaman B, Uluğ AV, et al. The association between prolongation in QRS duration and presence of coronary collateral circulation in patients with acute myocardial infarction. JRSM Cardiovasc Dis. 2016;5:2048004016657475–2048004016657475.

42. Baruah S, Chaliha MS, Borah PK, Rajkakati R, Borua PK, Mahanta J. Insertion/insertion genotype of angiotensin I-converting-enzyme gene predicts risk of myocardial infarction in northeast India. Biochem Genet. 2016;54:134–46.

43. Zhao W, Ma ST, Cui LQ. Meta-analysis of angiotensin-converting enzyme insertion/deletion polymorphism and myocardial infarction in Han Chinese. Genet Mol Res. 2015;14:8068–76.

44. Vergaro G, Fatini C, Sticchi E, Vassalle C, Gensini G, Ripoli A, et al. Refractory hyperaldosteronism in heart failure is associated with plasma renin activity and angiotensinogen polymorphism. J Cardiovasc Med. 2015;16:416–422.

45. Bezgin T, Kaymaz C, Akbal Ö, Yılmaz F, Tokgöz HC, Özdemir N. Thrombophilic gene mutations in relation to different Manifestations of venous thromboembolism: A single tertiary center study. Clin Appl Thromb Hemost. 2018;24:100–6.

46. Bargahi N, Ghorbian S, Akbar A, Zonouzi P, Poursadegh A. Genetic susceptibility to deep venous thromboembolism: The roles of inherited thrombophilia polymorphisms. Blood Coagul Fibrinolysis. 2016;27:308–12.

47. Han C, Han X-K, Liu F-C, Huang J-F. Ethnic differences in the association between angiotensin-converting enzyme gene insertion/deletion polymorphism and peripheral vascular disease: A meta-analysis. Chronic Dis Transl Med. 2017;3:230–41.

48. Evangelista FCG, Rios DRA, Ribeiro DD, Carvalho MG, Dusse LMS, Fernandes AP, et al. Lack of association between potential prothrombotic genetic risk factors and arterial and venous thrombosis. Genet Mol Res. 2015;14:9585–94.

49. Li Z, Wang S, Jiao X, Wei G. Genetic association of Angiotensin-Converting Enzyme I/D polymorphism with intracranial hemorrhage: An updated meta-analysis of 39 case-control studies. World Neurosurg. 2020;134:e1–7.

50. Angelidis G, Samara M, Papathanassiou M. Impact of renin-angiotensin-aldosterone system polymorphisms on myocardial perfusion: Correlations with myocardial single photon emission computed tomography-derived parameters. J Nucl Cardiol. 2018;26:1298–308.
